# Rapid determination of influenza vaccine potency by an SPR-based method using subtype or lineage-specific monoclonal antibodies

**DOI:** 10.3389/fimmu.2023.1128683

**Published:** 2023-06-29

**Authors:** Kartik Narayan, Crina Paduraru, Taylor Blake, Arun B. Arunachalam

**Affiliations:** Analytical Sciences, Vaccine R&D, Sanofi, Swiftwater, PA, United States

**Keywords:** surface plasmon resonace (SPR), influenza vaccine, hemagglutinin (HA), alternative potency assay, monoclonal antibodies

## Abstract

Potency testing and release of annual influenza vaccines require preparation, calibration, and distribution of reference antigens (RAs) and antisera every year, which takes an average of 8 to 12 weeks, and can be a major limiting factor in pandemic situations. Here we describe for the first time a robust Surface Plasmon Resonance (SPR)-based method that employs influenza subtype or lineage hemagglutinin (HA) specific monoclonal antibodies (mAbs) to measure the HA concentration in influenza multivalent vaccines. Implementing such an advanced test method will at the very least eliminate the rate-limiting and laborious efforts of making antisera reagents annually, and thus expedite the influenza vaccine delivery to the public by at least 6 weeks. Results demonstrate that the SPR-based method, developed using Biacore, is robust and not influenced by the type of RAs (inactivated whole virus, split, or subunit vaccine-derived materials), whether they are used as monovalent or multivalent preparations. HA concentrations obtained for monovalent drug substances (DS) or quadrivalent drug products (DP) of inactivated influenza split vaccine showed a tight correlation (the best fit value for the slope is 1.001 with R^2^ of 0.9815 and P-value <0.0001) with the corresponding values obtained by the current potency assay, Single Radial Immunodiffusion (SRID). Supplementary analysis of the results by the Bland-Altman plot demonstrated good agreement between the SPR and SRID methods, with no consistent bias of the SPR versus SRID method. We further demonstrate that the SPR-based method can be used to estimate HA concentrations in intermediates of the influenza vaccine manufacturing process containing varying matrices and impurity levels. Further, the results demonstrate that the method is sensitive to detecting degradation of HA caused by elevated temperature, low pH, and freezing. It is evident from this report and other published work that the advancement of analytical techniques and the early findings are encouraging for the implementation of alternate potency assays with far-reaching benefits covering both seasonal and pandemic influenza.

## Introduction

The seasonal influenza epidemic continues to be the major respiratory disease, causing severe illness and deaths in young children and elderly populations worldwide (1–3). Such a high disease incidence significantly burdens both social and economic costs, which underscores the timely availability of influenza vaccines to the public. Also, transmission and adaptation of influenza A virus strains from domestic animals to humans led to global pandemics in the last 100 years (4). Many government initiatives support the production and stockpiling of vaccines against such pandemic strains for emergency use in potential future pandemics.

Several influenza vaccines such as inactivated, live attenuated or recombinant vaccines are licensed and available globally (5). Most of the seasonal vaccines are quadrivalent comprising influenza A subtypes H1N1 and H3N2, and influenza B lineages B/Yamagata and B/Victoria. The annual production of influenza vaccine is arduous, and a race-against-time and timely availability of the seasonal influenza vaccine is critical for the completing vaccine campaign before the influenza epidemic peaks. More importantly, the supply of vaccines at an unprecedented ‘warp speed’ is critical to diffusing pandemic risk and potential public health emergencies (6, 7).

Two major proteins expressed by enveloped influenza virus are hemagglutinin (HA) and neuraminidase (NA). HA binds to its receptor, sialic acid on target cells and facilitates virus entry to the host cell and later on mediates the fusion of the viral envelope to the late endosomal membrane. Both these steps are critical for virus infectivity and thus, antibodies that block HA effectively prevent viral entry into target cells and thus, protect the host from infection (8, 9). HA being the primary protein in the induction of a protective immune response against the influenza virus, it is included as the core antigen in the vaccine and its concentration is measured to determine the vaccine potency.

Single Radial Immunodiffusion (SRID) has been used since the late 1970s as the potency assay for the release and stability testing of influenza vaccines. *Wood and Weir* have eloquently covered the historical perspective of SRID method development and implementation in detail (10). Originally, chicken cell agglutination (CCA) assay with an international standard was used to measure the potency of live attenuated virus vaccines which was found not suitable for split and subunit vaccines developed later (11–15). Fortunately, the SRID method developed in that time frame was found to have a gross correlation between HA content measured by the SRID method and the clinical immunogenicity (14, 15) for both vaccines containing either whole or disrupted virus. Later, SRID was further optimized with purified HA preparation and HA-specific hyperimmune antisera raised in goats (16). Following optimization of the method and implementation of calibrated reference standard and antiserum, the SRID method was accepted by the EU Committee for Medicinal Products for Human Use (CHMP) as the official potency method for releasing influenza vaccines for the European Market (17).

The SRID method measures HA content in vaccines based on a specific antigen-antibody reaction that forms a visible precipitin ring in the stained gel. The size (diameter) of the ring is directly proportional to the antigen concentration, which is calculated relative to a reference antigen with known potency. Although the SRID method measures the antigenicity of HA and is mostly specific to influenza strains tested, it relies on an outdated gel diffusion technique and critical reagents produced annually. It also suffers from poor reproducibility due to potential subjectivity in reading the precipitin rings. SRID is a labor-intensive method with low throughput making it not suitable for expedited vaccine release. Further, SRID has low sensitivity and is not suitable for testing flu vaccines with lower HA content (as low as 1.9 μg/dose) prepared for antigen dose-sparing pandemic clinical studies (18).

The annual manufacture of influenza vaccine is time-consuming and difficult. The production process, from the selection of influenza strains to vaccine manufacture and release for distribution, takes eight to nine months each year (6). One of the activities that takes a substantial amount of time is the generation of calibrated reference reagents for the potency assay, SRID. Strain-specific RAs and antisera are prepared annually, at a minimum, for the new strains included in the vaccines. Production and calibration of RAs by Essential Regulatory Laboratories (ERLs) are laborious and time-consuming (10, 19). Similarly, the generation and calibration of antiserum take around 8 weeks as it involves purification of HA, immunization of sheep, and qualification of antiserum for titer and specificity. In some years, difficulty in obtaining purified HA and poor immunogenicity of HA from certain strains led to the unavailability of these reagents on time for testing (10). Moreover, the use of animals for the generation of SRID reagents is a deviation from the ongoing 3Rs (Reduction, Refinement, and Replacement of animal usage) effort. All these shortfalls in making the annual reference reagents further limit the already burdened influenza manufacturing process. The availability of critical reagents for the potency assay can be a major limiting factor in pandemic situations. It can also delay the release of annual seasonal influenza vaccines for some years, which can potentially defer immunization well into the midst of the influenza epidemic. Hence, the US government made a recommendation to shorten the time and increase the reliability of the preparation of reagents for potency testing to expedite the influenza vaccine delivery (20).

The ideal solution to these rate-limiting and laborious efforts is to have perennial reagents. Sialic acid (SA) receptor is one such perennial reagent touted as a replacement for the current polyclonal antisera. SA receptor is the natural target molecule for the virus binding to the host cell and hence SA receptor-based assays would preferentially quantitate HA in the native conformation. However, one of the major drawbacks of the SA receptor is that it is not strain or even subtype-specific and therefore, SA receptor-based assays are not suitable for the quantitation of different strains of HA in multivalent vaccine preparations. Also, SA receptors need to be in multivalent formats for high-affinity and more stable interactions with HA rosettes in vaccine preparations (21). Because of its universal specificity across all strains of HA, the SA receptor is suitable only for the quantitation of monovalent vaccine preparations such as the pandemic vaccine. Another type of perennial reagent is mAb with subtype (for A strains) or lineage (for B strains) specificities. They can be generated using various technologies ranging from conventional hybridoma to modern recombinant technologies (22, 23). The availability of influenza subtype or lineage-specific antibodies will facilitate the use of the same set of antibodies every year even with strain change. Food and Drug Administration (FDA) with support from Biomedical Advanced Research and Development Authority has developed such subtype-specific mAbs (10, 24–26). In conclusion, the availability of all such perennial reagents facilitates the development of alternate potency assays for the expedited delivery of influenza vaccines annually.

Various technologies, using immunological reagents, have been successfully employed for the development of quantitative methods for protein or polysaccharide antigens. They include plate-based immuno-enzymatic or -fluorometric and multiplex techniques such as ELISA, Meso Scale Multi-array, Luminex, and biosensor technologies such as Surface Plasmon Resonance (SPR), and Bio-layer interferometry (BLI). Certain techniques are preferred for the quantitation of antigens based on their suitability. Both biochemical and immunoassays have been described as a replacement for SRID for measuring influenza potency. These include biochemical assays such as reverse-phase high-performance liquid chromatography (RP-HPLC) and Mass spectrometry (MS) and immunoassays based on SPR (such as Biacore), enzyme-linked immunosorbent and multiplex (such as VaxArray) technologies [reviewed by Woods and Weir (10)]. However, immunoassays that use functional antibodies or receptors are preferred over biochemical methods as they measure biologically relevant forms of HA. A few groups have reported the development of immunoassays for the quantitation of HA in vaccines and each one of them has its advantages and disadvantages (26–33).

Both biosensor and plate-based immunoassays have been described for the quantitation of HA and both can rapidly test a large number of samples. However, a few unique features of each platform bring out clear advantages, allowing one to be chosen over the other platform under certain situations. Plate-based techniques such as ELISA have been well-established for a variety of antigens and provide high throughput analyses. On the other hand, biosensor technology offers a label-free real-time measurement of antigen and antibody interactions and their quantitation as it utilizes real-time association and disassociation kinetics for analyte quantitation. This enables quick screening and selection of essential reagents and assay conditions, which is critical for the timely testing and release of influenza vaccines for each season. Biosensor platforms, such as Octet with parallel monitoring, allow sample testing up to a 384-well format, facilitating testing and analysis of a substantial number of samples with minimal supervision. In conclusion, biosensor-based assays would produce high-quality data, allow high throughput testing, and can be adapted rapidly (34). The biosensor platform is therefore suitable for both exploratory and official release testing.

Here we present an SPR-based approach that uses influenza subtype or lineage hemagglutinin (HA) specific mAbs to quantify the HA content in influenza vaccines. Results demonstrate that the SPR-based method is robust to accommodate different types of RAs and suitable for testing intermediates of influenza vaccine manufacturing process in different matrices. More notably, results showed a tight linear correlation between the SPR-based method and SRID covering all four strains both in monovalent DS or multivalent DP vaccine samples. The benefits of the SPR-based approach over SRID as well as its implementation strategy as an alternative potency assay are further described.

## Materials and methods

### Reference antigens and test materials

RAs were procured from one of the WHO-designated ERLs namely Center for Biologics Evaluation and Research (CBER; Rockville, MD, USA), NIBSC; Potters Bar, Hertfordshire, UK) or Therapeutic Goods Administration (TGA; TGA; Woden, ACT, Australia).

**Figure 1:**
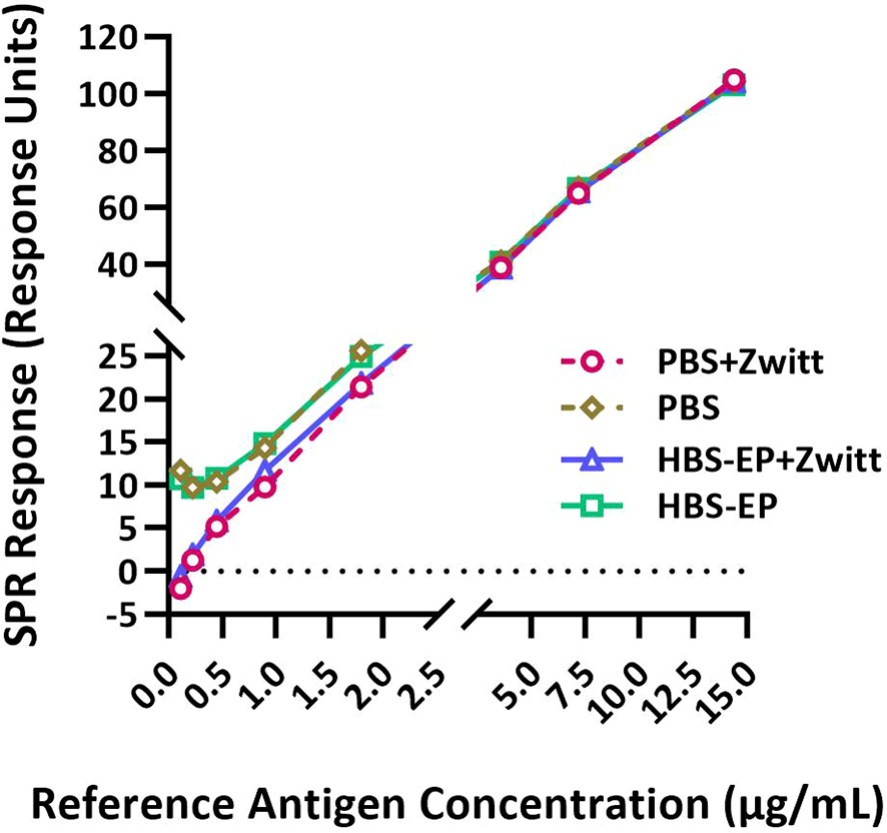
Zwittergent is necessary for RA and sample dilutions to achieve optimal sensitivity and linearity at lower concentrations. RA (B/Phuket/3073/2013) was disrupted with Zwittergent and then serially diluted in either PBS or HBS-EP+ with or without 1% Zwittergent and tested by the SPR-based method. SPR responses (in Response Units) obtained for RA diluted in different buffers plotted against HA concentrations are shown. Response curves obtained with different preparations are shown by solid or dashed lines with different symbols and colors as indicated in the figure labels. Both X and Y-axes are segmented to provide a magnified view of the lower part of the curves where they differ substantially.

**Figure 2:**
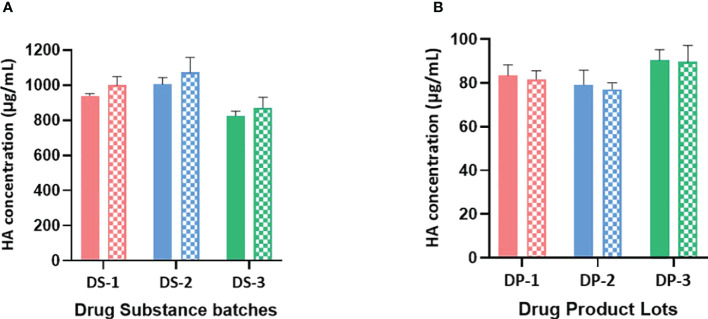
**(A)** B/Brisbane HA concentrations in drug substances are obtained using monovalent and bivalent RA preparations. HA concentrations in monovalent DS obtained using monovalent and bivalent RA preparations are shown in solid and patterned bars respectively. Monovalent RA preparation containing only B/Brisbane/60/2008 (B-V) or bivalent RA preparation containing both B/Brisbane/60/2008 (B-V) and B/Phuket/3073/2013 (B-Y) were prepared to contain 10μg/mL of each HA antigen. Both preparations were then serially diluted and used in the test. Error bars represent standard deviations of triplicate results. **(B)** B/Brisbane HA concentrations in drug products are obtained using monovalent and bivalent RA preparations. HA concentrations in quadrivalent DP obtained using monovalent and bivalent RA preparations are shown in solid and patterned bars respectively. Monovalent RA preparation containing only B/Brisbane/60/2008 (B-V) or bivalent RA preparation containing both B/Brisbane/60/2008 (B-V) and B/Phuket/3073/2013 (B-Y) were prepared to contain 10μg/mL of each HA antigen. Both preparations were then serially diluted and used in the test. Error bars represent standard deviations of triplicate results.

**Figure 3:**
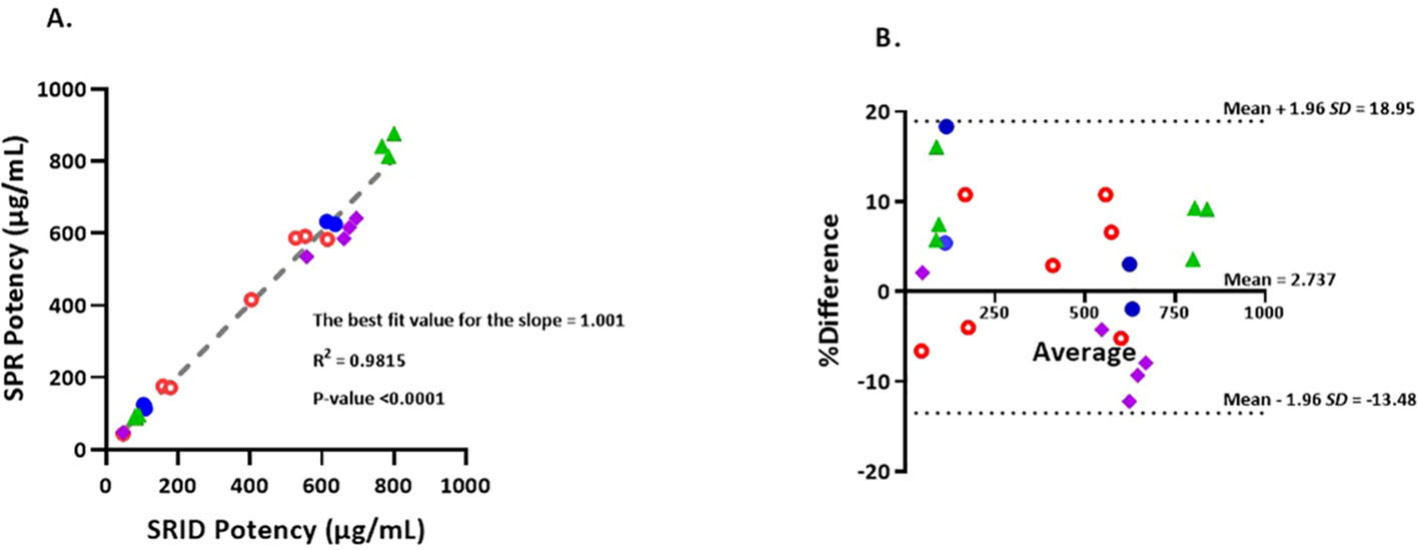
**(A)** Correlation of potency values obtained by SRID vs SPR-based method. Simple linear regression of SRID potency values against SPR potency values in µg/mL. Values that are below 200 µg/mL are from Fluzone drug product lots and values that are 400 µg/mL and above are from Fluzone DS lots. Blue closed circles represent A/Michigan (H1N1) potencies, Green triangles represent A/Hong Kong (H3N2) potencies, Red open circles represent B/Maryland (Victoria lineage) potencies and Purple squares represent B/Phuket (Yamagata lineage) potencies. **(B)** The Bland-Altman plot of agreement between SPR and SRID methods. %Differences between each pair of results (µg/mL) obtained by the two methods are plotted against the corresponding average values. The %difference is calculated using equation, 100*(SPR result – SRID result)/average. The 95% ‘limits of agreement’ is shown as dotted lines. Blue closed circles represent A/Michigan (H1N1) potency results, Green triangles represent A/Hong Kong (H3N2) potency results, Red open circles represent B/Maryland (Victoria lineage) potency results and Purple squares represent B/Phuket (Yamagata lineage) potency results.

**Figure 4:**
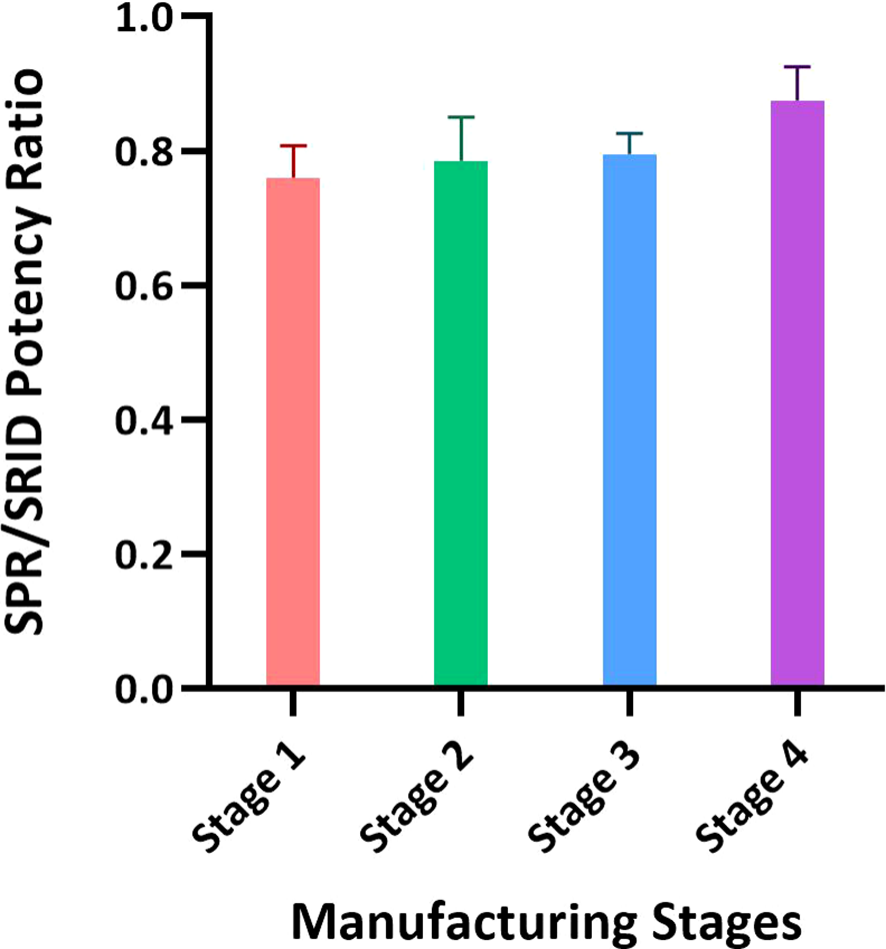
Application of the SPR-based method for testing materials from different stages of the influenza vaccine manufacturing process. Intermediates of the manufacturing process representing the crudest to the purest materials with the lowest to the highest HA concentrations were tested by SRID and the SPR-based method for HA concentration. Intermediates from three batches of A/Hong Kong (H3N2) and one batch of A/Kansas (H3N2) were tested and the ratios of average potency values obtained by these methods with each intermediate are shown. Each bar represents averaged SPR over SRID results for the same stage of four different batches. Error bars represent the standard deviation for each intermediate material tested.

Inactivated split influenza vaccine materials used in the development of the SPR-based method (**Figure 1**, **Tables 1**, **2**) and to demonstrate the method attributes (**Figures 2**–**4**) were obtained at various stages of the Fluzone manufacturing process including intermediates, DS and drug product. Additional details of the material used are provided in the results section.

**Table 1:** Effect of flow rate on the binding of drug substance samples to specific mAbs.

Sample	Flow rate (µL/min)	Binding of 150X dilution (RU)	Binding of 300X dilution (RU)	Binding of 600X dilution (RU)
**A/Michigan/45/2015 DS**	30	744.2	606.7	467.2
	50	750.3	609.9	475.2
	100	750.2	612.3	479.4
**A/Hong Kong/4801/2014 DS**	30	252.2	161.8	98.6
	50	254.4	163.2	99.5
	100	255.4	164.7	100.9
**B/Brisbane/60/2008 DS**	30	235.0	159.2	103.3
	50	231.0	154.9	99.8
	100	222.3	147.4	93.5
**B/Phuket/3073/2013 DS**	30	333.9	236.4	159.8
	50	333.8	234.2	157.1
	100	329.2	229.7	152.3

**Table 2:** Reactivity of mAbs to strains within a subtype or lineage of influenza virus.

A. mAbs tested against H1 RA				
mAbs	A/Brisbane/ 2/2018	A/California/ 07/2009	A/Michigan/ 45/2015	A/Guangdong-Maonan/ SWL 1536/2019
Anti-H1	23.3*	19.9	24.3	22.0
Anti-H3	0.2	0.2	0.3	NT
Anti-B/Victoria	0.7	0.5	0.0	NT
Anti-B/Yamagata	1.2	1.0	0.8	NT

**Table:** 

B. mAbs tested against H3 RA						
mAbs	A/Hong Kong/ 4801/2014	A/Kansas/ 14/2017	A/Singapore/ INFIMH-16-0019/2016	A/Switzerland/ 9715293/2013	A/Texas/ 50/2012	A/Victoria/ 361/2011
Anti-H1	0.3	0.3	0.2	0.3	0.3	0.3
Anti-H3	5.8	7.7	5.9	7.6	7.0	6.7
Anti-B/Victoria	0.0	0.0	0.0	0.7	0.5	0.1
Anti-B/Yamagata	0.4	0.0	0.0	1.2	1.1	0.9

**Table:** 

C. mAbs tested against B/Victoria RA			
mAbs	B/Brisbane/ 60/2008	B/Maryland/ 15/2016	B/Washington/ 02/2019
Anti-H1	0.3	0.3	0.2
Anti-H3	0.2	0.2	0.2
Anti-B/Victoria	4.4	3.6	0.0
Anti-B/Yamagata	0.5	0.0	0.0

**Table:** 

D. mAbs tested against B/Yamagata RA			
mAbs	B/Massachusetts/ 02/2012	B/Phuket/ 3073/2013	B/Texas/ 06/2011
Anti-H1	0.2	0.3	0.2
Anti-H3	0.1	0.1	0.1
Anti-B/Victoria	0.6	0.3	0.0
Anti-B/Yamagata	14.4	14.0	12.6

Values presented in this table are %Relative Surface Binding of RAs to subtype or lineage-specific mAbs.

NT, Not Tested.

Rows highlighted in green show the reactivity (i.e., specificity) of the selected monoclonal antibodies to specific strains within the relevant influenza subtype/lineage.

**Figure 5:**
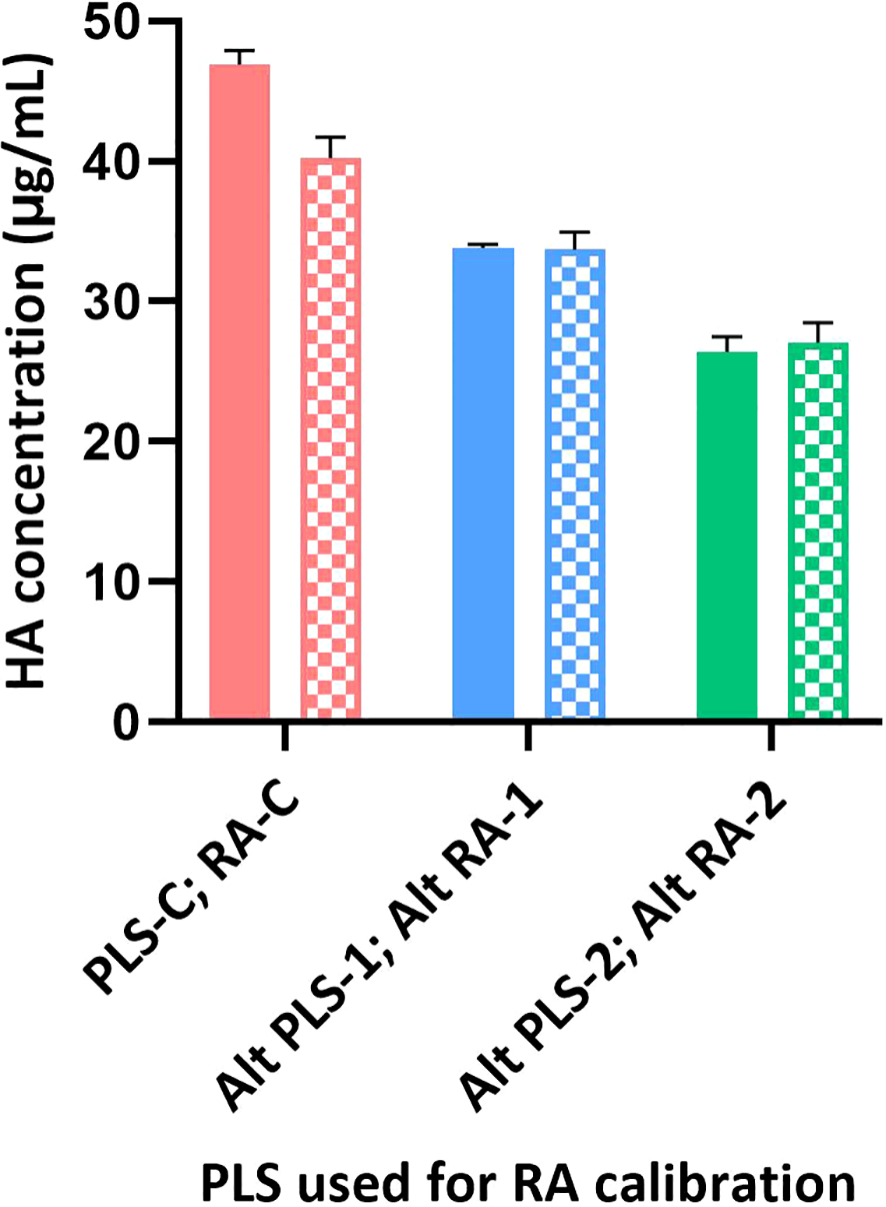
Calibration of different RAs using SRID and SPR-based methods. Different RAs (H1N1; A/Brisbane/02/2018) were calibrated against their corresponding PLS using SRID by ERLs and using the SPR-based methods by us. Calibrated values obtained for each RA by SRID (mean values from 3 ERLs) are shown as solid bars and by SPR-based method (mean values from triplicates) are shown as patterned bars. Error bars represent the standard deviation for each RA calibrated by SRID or SPR-based method.

**Figure 6:**
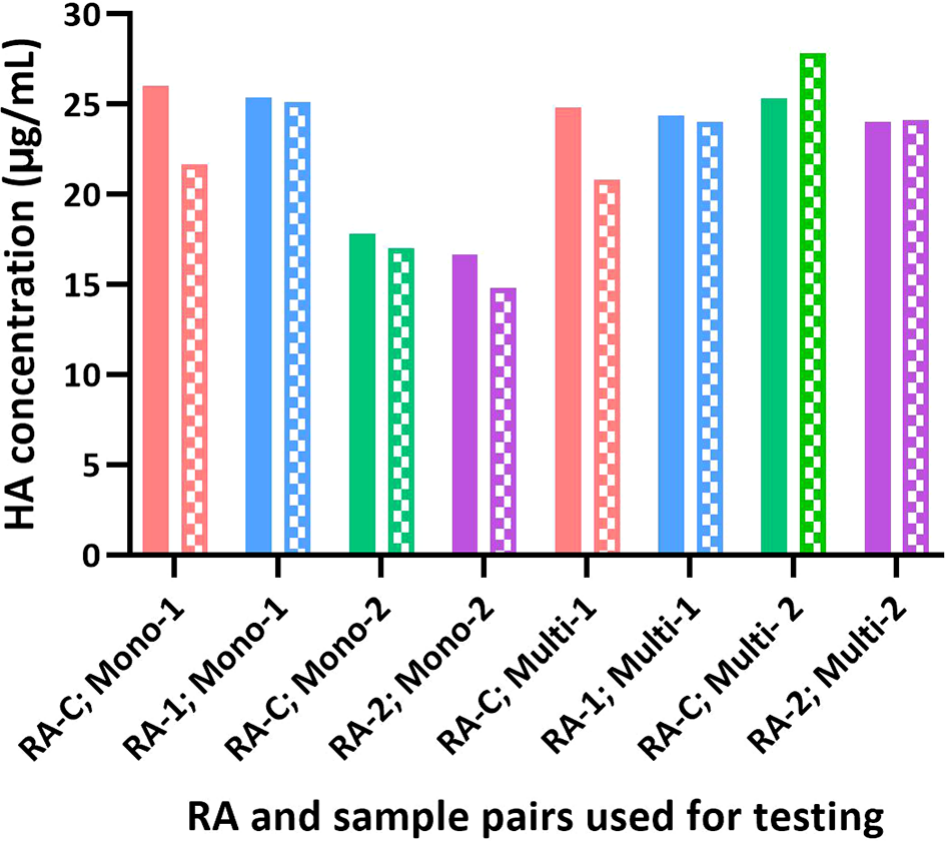
Influence of different RAs on the potency of test articles by SRID and SPR-based methods. The potency value assigned for each RA based on SRID was used to test H1 HA in the monovalent and multivalent test samples. The potency of each sample obtained by SRID (mean values from 3 ERLs) is shown as solid bars and by the SPR-based method (mean values from triplicates) is shown as patterned bars.

Results provided in **Figures 5**, **6** were generated as a part of a collaborative study between vaccine manufacturers, who are members of the International Federation of Pharmaceutical Manufacturers & Associations (IFPMA), the WHO’s ERLs, assay developers, and other interested stakeholders, to improve readiness for an influenza pandemic. The collaborative study investigated the use of alternative RAs in alternative potency assays. Monovalent and quadrivalent vaccine materials were used as test articles and the corresponding RA and Primary Liquid Standard (PLS) were provided by two influenza vaccine manufacturers. They were either split (Manufacturer-1) or subunit (Manufacturer-2) vaccine materials prepared from egg-propagated influenza virus that had been inactivated with formaldehyde. ERLs assigned the HA (H1N1; A/Brisbane/02/2018) concentration of each PLS and the potency value of each RA following the same conventional procedure used for generating official RAs annually. In brief, PLSs (PLS-C [PLS conventional], Alt PLS-1 [split material from Manufacturer 1], and Alt PLS-2 [subunit material from Manufacturer 2]) were tested by SDS-PAGE and total protein content and the amount of HA in each PLS was quantified by 3 ERLs. Values obtained for each PLS from ERLs were averaged and assigned to that PLS. Then RAs (RA-C [RA conventional], Alt RA-1 [split material from Manufacturer 1], and Alt RA-2 [subunit material from Manufacturer 2]) were calibrated against their corresponding PLS by SRID and potency values from 3 ERLs were averaged and assigned to each RA. The calibrated RAs (RA-C, Alt RA1, or Alt RA2) were used in SRID and the SPR-based method to test HA antigen (H1N1; A/Brisbane/02/2018) in monovalent and multivalent samples obtained from the manufacturers 1 & 2. Additional details on PLS, RA, and test materials used in this study are provided in **Table 3**.

**Table 3:** Materials tested for assessing the use of alternative reference antigens.

Source	PLS (H1N1; A/Brisbane/02/2018) used for calibration	Reference Antigen (H1N1; A/Brisbane/02/2018) calibrated	Test Articles^§^ (Monovalent/Multivalent)
Manufacturer 1 or 2 (blinded)	PLS-Conventional (PLS-C) Inactivated whole virus	RA-Conventional (RA-C) Inactivated whole virus, lyophilized	Mono -1 or Multi-1 Inactivated split material Mono-2 or Multi-2 Inactivated subunit material
Manufacturer-1	Alt PLS-1 Inactivated split material	Alt RA-1 Inactivated split material, lyophilized	Mono-1 & Multi-1 Inactivated split material
Manufacturer-2	Alt PLS-2 Inactivated subunit material	Alt RA-2 Inactivated subunit material, lyophilized	Mono-2 & Multi-2 Inactivated subunit material

### Monoclonal antibodies

H1N1 subtype-specific human mAb (5J8) is published in the literature (35). H3N2 subtype-specific mAb and its specificity are described in the results section. Both B/Victoria-lineage (B-V) specific mouse mAb (BR8E12) and B/Yamagata-lineage (B-Y) mouse mAb (WI3E8) were kindly provided by Dr. Jerry P Weir, Division of Viral Products, FDA, USA (26). Antibodies were stored in Eppendorf tubes at -70°C as single-use 10μL aliquots. For each experiment, one aliquot is diluted to 10 μg/mL in HEPES Buffered Saline supplemented with EDTA and 0.05% Tween 20 (HBS-EP+).

### SRID procedure

SRID assay was performed in ERL (NIBSC, CBER, and TGA) and our laboratory following the basic procedure described previously by *Wood et al.* (16) with modifications as described in the later publications (36, 37). In brief, gels were cast with 1% agarose in PBS containing the optimal amount of strain-specific antiserum. RAs treated with Zwittergent were distributed into wells that had been punched into the gel. Zwittergent‐treated samples were serially diluted and loaded onto the agarose gel and the gels were then incubated at room temperature for 18–24 hours in a humidified chamber to allow the antigens to diffuse. Following incubation, gels were washed, dried, and stained with Coomassie Brilliant Blue. Diameters of the precipitin ring on the stained gel were measured and used to generate a dose-response curve against a RA of pre‐determined HA concentration. The concentration of HA (i.e., potency) in the sample was calculated from the linear region of the parallel dose-response curve and expressed in μg/mL.

### SPR-based test procedure

Cytiva/GE Biacore T100 instruments and CM5 Series S sensor chips were used for the development of a direct binding assay and testing of various samples. In this SPR-based method, the antibody was captured onto a Protein A/G surface of a sensor chip. A calibration curve was generated by injecting split RA at known concentrations over the antibody-captured surface. Specific dilutions of unknown samples were then injected over the antibody-captured surface and residual binding was measured. From this, a calibration curve was generated using a non-linear fit. Based on the response of the unknown sample (and where it fell on the reference standard curve) and the dilution factor, a potency value for the test sample was calculated. Each injection had a flow rate of 30 μL/min with a contact time of 60 seconds and a dissociation time of 60 seconds. HA concentration in test samples is expressed in µg/mL. A more detailed description of the method follows.

First, the Protein A/G (Pierce Cat # 21186) was diluted to 40 µg/mL in acetate buffer with pH 4.5 (Cytiva/GE Cat # BR-1003-50) and immobilized to CM5 Series S sensor chip (Cytiva/GE Cat # 29149603) using standard EDC/NHS coupling for 8 minutes on each flow cell (38). Excess reactive groups were deactivated by injecting ethanolamine for 6–7 min. Typical immobilization amounts ranged from 2300 – 2800 response units (RU) per flow cell. Subtype/lineage-specific mAbs were diluted to 10 µg/mL in 1x HBS-EP+ (Cytiva BR100669 (HEPES Buffered Saline supplemented with EDTA and 0.05% Tween 20, 10x). Specific antibody per each flow cell was captured at a flow rate of 10 μL/min with a contact time of 30 seconds, except Flow Cell 1 which was used for reference. Lyophilized RA obtained from an ERL was dissolved in water and then diluted to 40 µg/mL in HBS-EP+. 10% Zwittergent 3-14 (EMD Millipore Cat # 693017-5GM or equivalent grade) was added to the RA preparation to obtain a final 1% Zwittergent concentration and incubated for 30 minutes at room temperature with end-over-end rocking. RAs were prepared either as a monovalent or multivalent by combining different strains while maintaining the final concentration of HA at around 40 µg/mL and of Zwittergent at 1%. Two-fold serial dilutions of RA were prepared in HBS-EP+ buffer containing 1% Zwittergent (HBZ buffer), in a polypropylene 96-well plate (Greiner Bio-one, 650201) to generate a 10-point calibration curve ranging from 10 µg/mL to 0.0195 µg/mL. Similarly, two-fold dilutions of test samples were prepared using HBZ buffer to generate 6 dilutions/sample. RA and samples were injected at a flow rate of 30μL/min with a contact time of 60 seconds and dissociation time of 60 seconds. Regeneration was carried out using 50 mM NaOH (Cytiva/GE Cat # BR-1003-58 or equivalent grade) with 10 seconds contact time and 10 µL/min flow rate.

### Data analysis

Samples were analyzed using the Concentration Analysis feature of the Biacore T200 Evaluation software. The RA calibration was generated using a 4-parameter non-linear fit of response units (RUs) versus HA concentrations. RUs were obtained after the beginning of the dissociation phase. A valid calibration curve had to have a minimum of 6 points, with Chi^2^ value of the 4 parameters fit less than 1% of R_hi_. Samples had to have a minimum of 3 dilutions within the calibration curve range and the corresponding dilution-corrected results had to have coefficient variation (CV) <20%. The sample dilution range was further refined if at least 3 dilutions did not fall in the RA calibration range. HA concentration in test samples was calculated as the average of valid sample dilutions and was expressed in µg/mL. The HA concentration (µg/mL) determined by the SPR method is referred to as SPR or alternate potency.

## Results

### Effect of Zwittergent on antigen-antibody binding

SRID was performed using 1% Zwittergent both to disrupt the RA and during sample preparation. RAs were prepared as directed in the circular provided by the respective ERL. To determine if Zwittergent is required for the calibration curve, the Zwittergent disrupted RA (B/Phuket/3073/2013) was further diluted in either PBS or HBS-EP+ with or without 1% Zwittergent and tested in the SPR-based method. Representative results shown in **Figure 1** suggested that Zwittergent at HA concentrations above 2µg/mL did not affect RA binding to the lineage-specific mAbs. However, at HA concentrations below 2µg/mL, Zwittergent increased the sensitivity of binding which resulted in a more linear response (**Figure 1**). Without Zwittergent, the binding of RA artificially bottomed at 0.5 μg HA/mL and thus, RA at lower concentrations and in the presence of Zwittergent demonstrated dose-dependent binding. There was no considerable difference in binding between HBS-EP+ and PBS. Thus, to achieve the desired sensitivity at lower concentrations, it is recommended to add Zwittergent to the buffer used for both RA and sample dilution preparations.

### Effect of flow rate on the antigen binding response

To determine if the flow rate is important in sample response, DS from four strains (A/Michigan/45/2015 [H1N1]; A/Hong Kong/4801/2014 [H3N2]; B/Brisbane/60/2008 [B-V] and B/Phuket/3073/2013 [B-Y]) were injected at three dilutions (150X, 300X and 600X) and three flow rates (30, 50 and 100 μL/min) over the specific mAbs surfaces and the binding responses, expressed as RU, were determined. Binding responses were comparable between three flowrates tested within each dilution (**Table 1**), which suggests that the flow rate is not an important criterion if it is kept constant throughout the entire run, for both calibration and unknown samples. For subsequent experiments and testing, a flow rate of 30 μL/min with a contact time of 60 seconds was chosen to conserve the sample without affecting HA concentration determination.

### Specificity of mAbs

To assess the specificity of the mAbs used in this study, RAs of different strains within each subtype or lineage were diluted to 10 µg/mL and injected over each mAb surface. The binding response was measured, and a % Relative Surface Binding value was calculated. This was performed by determining the theoretical response assuming that all the mAb and sample were capable of complete interaction in a 1:1 model and comparing the actual RA binding value. Results demonstrate that the mAbs used in this study are subtype (H1 or H3) or lineage (B/Victoria [B-V] or B/Yamagata [B-Y]) specific with broad binding to strains tested within each subtype or lineage without cross-reactivity to other subtype or lineage (**Table 2**). H1 specific mAb (5J8) showed optimal binding to four different H1 strains tested (**Table 2A**). Similarly, H3 specific mAb showed optimal binding to six different H3 strains tested (**Table 2B**). B-V lineage-specific mAb (BR8E12) showed optimal reactivity with two of three B-V strains tested, indicating that additional mAbs need to be screened and included in the assay for greater coverage within this lineage (**Table 2C**). B-Y lineage-specific mAb (WI3E8) reacted equally well with three different strains tested within the B-Y lineage (**Table 2D**).

### Monovalent versus bivalent reference antigen binding

The selected antibodies are subtype or lineage-specific and did not show observable cross-reactivity between subtypes or lineages. So, it is possible to use mixed RAs instead of monovalent RA to generate a calibration curve. To assess the possibility of using a mixed RA, three lots of monovalent DS and three lots of quadrivalent DP were tested for B/Brisbane HA concentrations using a bivalent (B/Brisbane/60/2008 [B-V] and B/Phuket/3073/2013 [B-Y] RAs mixed) and a monovalent (B/Brisbane) RA preparations. To prepare the bivalent RA preparation, each RA was disrupted with Zwittergent at 40 μg/mL of HA concentration and then combined to achieve 10μg/mL of HA per each antigen, which was then serially diluted and tested. Results suggested that B/Brisbane HA concentrations obtained for DS and DP using bivalent and monovalent RA preparations are comparable (**Figure 2**) suggesting that both monovalent and bivalent RA preparations could be used interchangeably. A similar outcome was obtained for B/Phuket using monovalent and bivalent RA preparations (results not shown).

### Influence of various types of reference antigens on the potency measured

Sanofi has participated in collaborative studies evaluating various parameters of alternate potency assays developed by different Health Authorities (CBER and NIBSC), vaccine manufacturers (Sanofi and Seqirus), and related biotechnology companies (InDevR). One of the studies evaluated the influence of alternate RAs (H1N1; A/Brisbane/02/2018), that were calibrated by SRID or by the alternate assay, on the potency values of blinded monovalent or multivalent samples. Results presented here suggest that the SPR-based assay is tightly aligned with SRID across all types of RAs and samples assessed (**Figures 5**, **6**). HA concentrations obtained for different RAs when they were calibrated independently using SRID by ERLs and using the SPR-based method by us against their corresponding (i.e., homologous) PLS are shown in **Figure 5**. Regardless of the type of RA evaluated, calibration values obtained by these two methods are nearly the same for each RA, suggesting that these assays recognize HA in these RAs comprising entire virus, split antigens, or subunit antigens in the same manner. PLSs and RAs were supplied at different concentrations, and they were not adjusted to having a uniform concentration when they were prepared. So, the different HA concentrations shown for different PLSs or RAs do not reflect the assay evaluated. Since these assays were performed in the same way within each RA tested, we tested blinded monovalent and multivalent samples by SRID and the SPR-based method in parallel using conventional or homologous RA to validate our findings with different RAs shown in **Figure 5**. For this experiment, we used the RA calibration values obtained by SRID. Results showed that the HA concentrations of monovalent and multivalent samples by SRID and the SPR-based methods are well aligned, and the results differed between-17% and +10% with an average variability of 5% (**Figure 6**). It confirms the conclusion we had previously drawn from the calibrations of RAs using these two techniques.

### Correlation of potency values by the SPR-based method with the SRID method

To determine the level of correlation between SRID and the SPR-based methods, we tested 22 lots of Fluzone vaccine materials comprising both monovalent DS and multivalent DP with potency ranging from 40 μg/mL to 800 μg/mL covering H1, H3, B-Y & B-V strains. The potency results obtained by SRID and SPR-based methods were plotted and a linear regression analysis was performed (**Figure 3A**). A simple linear regression analysis of the results suggests that SPR-based method potency values correlate tightly with the corresponding SRID potency values. The best fit value for the slope is 1.001 with R^2^ of 0.9815 and P-value <0.0001. Further analysis, for agreements of the potency values by the two methods, using the Bland-Altman test demonstrates that the mean bias is 2.737 with a standard deviation (*SD*) of 8.273 and the 95% limits of agreement ranging from -13.48 to 18.95 (**Figure 3B**; 39). The points on the Bland-Altman plot are uniformly scattered both above and below the zero line and between the 95% limits of agreement, suggesting that there is no consistent bias of the SPR versus SRID method, and there is a good agreement between the SPR and SRID methods. Thus, the Bland-Altman plot verifies the tight correlation between the SPR and SRID methods concluded from the simple linear regression analysis.

### Suitability of the SPR-based method for testing intermediates of inactivated influenza split vaccine manufacturing process

To assess the suitability of the SPR-based method to test intermediates of the influenza vaccine manufacturing process we aliquoted samples covering upstream to downstream steps of the manufacturing process. Typically, the upstream samples have the lowest concentration of HA and high impurities, and the downstream samples have high concentrations of HA with lower impurities. The difference in the matrices and levels of impurities in these samples is known to interfere with several test methods. Results shown in **Figure 4** were obtained from testing 3 batches of A/Hong Kong (H3N2) and one batch of A/Kansas (H3N2); they are expressed as an average ratio of HA levels by the SPR-based method over SRID for each sample type. Stage 1 to Stage 4, shown in **Figure 4**, represent the most upstream to the most downstream samples in the influenza vaccine manufacturing process. Each bar in **Figure 4** represents averaged SPR over SRID results for the same stage of four different batches. Results suggest that the SPR-based method is not considerably impacted by these matrices and hence, it can be used to test both the upstream and downstream samples (**Figure 4**). It should be noted that even with SRID, the upstream samples generally have high variability. The correlation between the SPR-based method and SRID values improved steadily with purification steps, attaining the best correlation at the downstream stage (i.e., Stage 4), where the HA concentration is the highest and impurities are at the lowest levels among the sample types evaluated here.

## Discussion

Each batch of influenza vaccines is tested for potency before they are released to the market. The current potency assay for influenza vaccines is SRID, which was developed and introduced into use more than forty years ago (16). It takes a lot of effort and time to prepare and calibrate RAs and antisera specific to strains when new strains are introduced in vaccines for the northern or the southern hemisphere influenza seasons annually (10, 19). The supply of reagents in the past years has been further hampered by poor yield and the immunogenicity of some strains (10). All these shortcomings are likely to delay the release of seasonal influenza vaccines to the market before the influenza epidemic peaks. In pandemic scenarios, any delay in the availability of critical reagents for the potency assay can cause a major health catastrophe. One of the best approaches to circumvent this issue is to implement high throughput assays based on cutting-edge technologies that make use of enduring reagents with subtype or lineage pan-specificity, which can be used for many years with strain change. We chose to develop an SPR technology (Biacore) based alternative potency assay that applies label-free detection, provides real-time interaction details, and is highly sensitive to quantitate antigens at low concentrations.

The most critical element of any immuno-assay is the antibody, and the use of functional and conformation-sensitive antibodies would demonstrate the functionality and stability of the antigen quantitated. First, we screened several subtype or lineage-specific mAbs and identified the ones that reacted with most of the strains within a subtype or lineage and did not show any cross-reactivity between subtypes or lineages. The selected mAbs demonstrated pan-specificity within each subtype or lineage which corroborates observations reported by other groups (**Table 2**). *Krause et al.* found that the mAb 5J8 recognized HA from a wide range of H1N1 seasonal strains and 2009 pandemic H1N1 strain except A/New Caledonia/20/1999 or A/Brisbane/59/2007 (35). It inhibited hemagglutination and neutralized H1N1 viruses in a microneutralization assay and a lethal challenge mouse study. It was predicted from screening escape mutants and naturally occurring H1N1 strain that 5J8 mAb recognizes a novel conserved epitope on the globular head of HA that includes residues 133A, 137, and 222 (35, 40). We observed that both H1 and H3 specific mAbs exhibited reactivity only with strains of H1 and H3 subtypes respectively verifying their subtype pan-specificity (**Table 2**). Our findings demonstrating the lineage specificity of BR8E12 and WI3E8 antibodies in the SPR-based assay are consistent with the results reported in the literature. Verma et al, reported the specificities of BR8E12 to B/Victoria lineage and WI3E8 to B/Yamagata lineage using ELISA, hemagglutinin inhibition, and viral neutralization methods (26). Antibody BR8E12, specific to HA from the B/Victoria lineage, recognizes the globular head region of HA and is sensitive to the mutation of amino acid residue at 241 (P241Q) (26). MAb BR8E12 was generated to B/Brisbane/60/2008, a B/Victoria strain that preceded the emergence of the triple deletion (positions 162-164) B/Victoria viruses such as B/Washington/02/2019. Ferret antisera raised against B/Victoria lineage viruses with no amino acid deletion in the HA (i.e., B/Brisbane/60/2008) poorly inhibited viruses with the triple deletion (i.e., B/Washington/02/2019) (41). Therefore, it is not surprising that BR8E12 did not recognize the triple deletion mutant, B/Washington/02/2019. Likewise, mAb WI3E8 specific to HA from the B/Yamagata lineage recognizes the head region of HA and is sensitive to mutation of amino acid residue at 141 (G141E) (26). These mAbs were successfully used to develop an ELISA-based alternate potency assay that showed a good correlation over a wide range of HA concentrations with a regression fit slope of 0.94 and 1.75 for B-V and B-Y lineages respectively (26). In summary, the results reported by other groups and those generated in our lab demonstrate that the mAbs used in the SPR-based method have pan-specificity for strains within each subtype or lineage and they recognize functional epitopes on HA.

Our results suggested that the use of Zwittergent for the preparation of both RA and vaccine samples, including their serial dilution improved the response curve, especially at lower concentrations (**Figure 1**). It is likely that Zwittergent presence maintained homogeneous HA rosettes in the test articles and stabilized them at low concentrations. *Schmeisser et al.* made a similar observation demonstrating a better correlation between ELISA and SRID results when both standard and samples were treated with Zwittergent (32). Comparable binding responses with varying flow rates demonstrate that the binding is not mass transport limited (**Table 1**). Since the SPR method described here uses calibration curves to estimate HA concentrations in samples, it is not necessary to determine antigen-antibody interactions under mass transport limiting conditions. HA from B strains of B/Victoria and B/Yamagata lineages are antigenically closely related and exhibits considerable levels of cross-reactivity with polyclonal antisera generated against each other (26). Therefore, a bivalent reference-antigen preparation with equal levels of HA from both lineages is used for SRID testing to remove any potential bias in assessing the potency of B strains in quadrivalent preparations. The specificity of the mAbs utilized in the SPR-based assay is further confirmed by the comparable HA concentrations obtained for DS and DP lots using monovalent and bivalent RA preparations (**Figure 2**).

Some alternate methods have been found to respond differently based on the type of RA used. *Kuck et al.* reported that the VaxArray method generated different slopes of the calibration curve with ERL RA (lyophilized intact virus) and an internal RA (split virus) (42). Since both RAs were treated with Zwittergent to potentially generate comparable rosettes, they hypothesized that the difference in the degree of chemical modifications of RA could have resulted in differential binding observed in their assay (42). We explored conventional RA (whole virus), split vaccine as RA (Alt RA-1), and subunit vaccine as RA (Alt RA-2) in both SRID and SPR-based methods. Results showed that both methods generate comparable potency values for various types of samples tested (**Figure 6**). Also, the SPR-based method was applied in parallel to SRID for testing the crudest to the purest vaccine materials in the Fluzone vaccine manufacturing process. Allantoic fluids from influenza virus-propagated embryonated eggs are pooled in stage 1 of the manufacturing process. The HA concentration at this stage is expected to be around 1% of the total protein. Modern chromatographic methods cannot accurately or precisely quantitate HA concentrations at this stage, and it requires frequent column changes due to the nature of the matrix. Thus, SRID has been the only method used for HA quantitation at this upstream stage of the process. When the SPR-based method was used to determine the HA concentrations of A/Hong Kong and A/Kansas strains at these intermediate stages of the manufacturing process, there was a good agreement with SRID (**Figure 4**). Low concentrations of HA and high variability of the SRID method with upstream samples would have been partly responsible for the 20% bias observed between SPR and SRID results for materials harvested earlier in the manufacturing process (**Figure 4**). Results suggest that the SPR-based method, which is rapid and has high throughput could be applied effectively for testing manufacturing in-process materials.

*Bodle et al.* reported the development of an ELISA as an alternative to SRID for measuring the potency of influenza vaccines (28). The method was 10 to 50 times more sensitive than SRID in detecting HA and demonstrated a linear correlation with SRID when all strains were analyzed together. Nevertheless, each antibody set used in the assay recognized only a few strains within each subtype or lineage necessitating frequent replacement of antibodies and the development of ELISA. *Hashem et al.* described an ELISA method that used sialic acid-containing fetuin to capture HA and strain-specific polyclonal antiserum for the detection of HA (29). However, the level of SA on fetuin is likely to vary significantly between batches and vendors making the results inconsistent (30). Moreover, reliance on strain-specific polyclonal antiserum every year for this assay does not enable the timely release of vaccines to the market. To circumvent these issues, *Khurana et al.* developed an SPR-based method utilizing synthetic glycans containing α-2,6 or α-2,3 sialic acids (SA), which uses label-free detection and showed a tight correlation with SRID (30). However, this method can be used for testing only the monovalent materials as the SA receptor cannot distinguish HA from four different strains included in influenza vaccines (10). Additionally, pre-screening is needed to determine the optimal type of receptor as the affinity of HA generated from egg versus mammalian cell-derived vaccines for α-2,6 and α-2,3 SA receptors is likely to differ. The SPR-based method described here uses for the first-time influenza subtype or lineage HA-specific mAbs, which provides the required specificity for testing multivalent influenza vaccines and renders the assay independent of annual antisera reagents.

*Schmeisser et al.* observed that only a small percentage of the mAbs tried in their ELISA generated results that were ≤20% of the corresponding SRID values, emphasizing the significance of choosing appropriate antibodies for the alternate potency assay (32). For our SPR-based assay, we screened several mAbs for their specificity and suitability, and we selected the ones that displayed pan-specificity within a subtype or lineage and demonstrated an optimal correlation with SRID. Selected mAbs showed a broad reactivity with strains within the subtype or lineage and recognize protective epitopes in either globular head or fibrous stem regions of HA (**Table 2**). Results from the SPR-based method, covering all four strains both in monovalent (i.e., DS) or multivalent (i.e., DP) vaccine samples, showed a tight linear correlation with results generated by SRID, with the best regression fit slope of 1.001 with R^2^ of 0.9815 and P-value <0.0001 (**Figure 3A**). Supplementary analysis of the results by the Bland-Altman plot demonstrated good agreement between the SPR and SRID methods, with no consistent bias of the SPR versus SRID method (**Figure 3B**). Limited epitopes recognized by the mAb in the SPR-based method may not truly represent multiple epitopes potentially recognized by the polyclonal serum used in SRID. Perhaps the antibodies in the polyclonal serum are predominantly against a few dominant epitopes on HA which along with the poor sensitivity of SRID likely brought the correlation linear and tight. The coverage of future strains would be increased by using a cocktail of mAbs for each subtype or lineage, which also would reduce the possibility of rejecting a vaccine batch based on the potential unavailability of a single epitope on HA. Moreover, the use of a cocktail of mAbs covering unique epitopes that are preserved only in the baculovirus-expressed recombinant HA (rHA) along with protective epitopes on the wildtype HA would allow monitoring those epitopes in the recombinant vaccines (reviewed by *Arunachalam et al.* (43)). Such an assay would be suitable for monitoring the potency of both wildtype virus-derived and recombinant influenza vaccines. Therefore, we are constantly screening for new mAbs, that can recognize distant non-overlapping epitopes both in wildtype and recombinantly expressed HA, for their inclusion in the SPR-based assay. The use of subtype or lineage-specific mAb cocktails eliminates the need for annual generation and qualification of strain-specific antisera, as well as re-validation of the potency method. Nevertheless, the specificity of the mAb cocktails for the new strains chosen for the influenza season must be verified annually.

Replicate results obtained for multiple strains from various experiments suggested that the SPR-based method is precise with an average Coefficient of Variation (CV) of 5.7% for repeatability and 6.0% for intermediate precision. Its estimated limit of detection (LOD) is around 5 ng/mL and its estimated limit of quantitation (LOQ) is around 40 ng/mL. Thus, the SPR-based method is around 150 times more sensitive than the SRID method (LOQ 6 μg/mL; observed in our lab). In addition, the SPR-based method is significantly faster than SRID; a monovalent sample can be tested, and results can be reported in less than 3 hours with the SPR-based method as opposed to 30 hours with SRID. As part of the collaborative effort, we recently completed a study evaluating the stability indicating the nature of alternate potency assays. Results demonstrated that the SPR-based method is highly sensitive to the degradation of HA induced by elevated temperature, low pH, and freezing (44).

In conclusion, the SPR-based method is robust to accommodate RAs and test materials of different matrices and HA concentrations. It showed a tight linear correlation with SRID over a wide range of strains and HA concentrations. The high specificity of the mAbs used in the SPR-based assay will also facilitate applying this method for vaccine monovalent DS identity testing for distinguishing them between subtypes and lineages. Hence, we believe that the SPR-based assay, similar to ELISA, is a promising replacement candidate for SRID for the release and stability testing of influenza vaccines. It would be prudent to demonstrate that alternative potencies are relevant in predicting the clinical efficacy (i.e., clinical immunocorrelate of protection for influenza). Such an endeavor needs clinical studies utilizing vaccines with varying levels of potency including subpotent materials. Certainly, such an effort requires ample resources, time, and a large number of clinical subjects for adequate statistical power. There is no doubt that the alternative potency approaches, like the SPR-based assay reported here, are critical for releasing pandemic influenza vaccines to meet future urgent medical demands.

## Data availability statement

The original contributions presented in the study are included in the article/supplementary material. Further inquiries can be directed to the corresponding author.

## Author contributions

KN, CP, and TB designed and conducted different experiments and analyzed the acquired data. AA conceptualized and prepared the original draft of the manuscript and the figures. All the authors reviewed and commented on the manuscript at all stages and remain accountable for the accuracy and integrity of the work. All authors had full access to all the data in the study and had final responsibility for the decision to submit for publication. All authors contributed to the article and approved the submitted version.

## References

[1] Iuliano AD Roguski KM Chang HH Muscatello DJ Palekar R Tempia S. Estimates of global seasonal influenza-associated respiratory mortality: a modelling study. Lancet (2018) 391:1285–300. doi: 10.1016/s0140-6736(17)33293-2 PMC5935243 29248255

[2] World Health Organization. Fact-sheet. influenza (Seasonal) (2018). Available at: https://www.who.int/en/news-room/fact-sheets/detail/influenza-(seasonal) (Accessed 12 March 2021).

[3] US Centers for Disease Control and Prevention (CDC). Weekly U.S. influenza surveillance report . Available at: https://www.cdc.gov/flu/weekly/index.htm (Accessed 15 December 2022).

[4] Paules C Subbarao K. Influenza. Lancet (2017) 390:697–708. doi: 10.1016/S0140-6736(17)30129-0 28302313

[5] Sparrow E Wood JG Chadwick C Newall AT Torvaldsen S Moen A. Global production capacity of seasonal and pandemic influenza vaccines in 2019. Vaccine (2021) 39(3):512–20. doi: 10.1016/j.vaccine.2020.12.018 PMC7814984 33341308

[6] Orenstein WA Schaffner W. Lessons learned: role of influenza vaccine production, distribution, supply, and demand–what it means for the provider. Am J Med (2008). 121:S22–27. doi: 10.1016/j.amjmed 18589064

[7] Weir JP Gruber MF. An overview of the regulation of influenza vaccines in the united states. Influenza Other Respir Viruses (2016) 10:354–60. doi: 10.1111/irv.12383 PMC4947948 27426005

[8] Hobson D Curry RL Beare AS Ward-Gardner A. The role of serum haemagglutination-inhibiting antibody in protection against challenge infection with influenza A2 and b viruses. Epidemiol Infect (1972). 70:767–77. doi: 10.1017/S0022172400022610 PMC2130285 4509641

[9] Ohmit SE Petrie JG Cross RT Johnson E. And monto A.S. influenza hemagglutination-inhibition antibody titer as a correlate of vaccine-induced protection. J Infect Dis (2011). 204:1879–85. doi: 10.1093/infdis/jir661 21998477

[10] Wood JM Weir JP. Standardisation of inactivated influenza vaccines-learning from history. Influenza Other Respir Viruses (2018). 12(2):195–201. doi: 10.1111/irv.12543 29356318 PMC5820418

[11] Hirst GK. The agglutination of red cells by allantoic fluid of chick embryos infected with influenza virus. Science (1941) 94:22–23. doi: 10.1126/science.94.2427.22 17777315

[12] Miller GL Stanley WM. Quantitative aspects of the red blood cell agglutination test for influenza virus. J Exp Med (1944) 79:185–195. doi: 10.1084/jem.79.2.185 19871362 PMC2135446

[13] WHO. 20th report of the WHO expert committee on biological standardization. WHO Tech Rep Ser. (1968) 384: 15–6. Available at: https://www.who.int/publications/i/item/9241203846 (Accessed 15 December 2022).

[14] Ennis FA Mayner RE Barry DW Manischewitz JE Dunlap RC Verbonitz MW. Correlation of laboratory studies with clinical responses to A/New Jersey influenza vaccines. J Infect Dis (1977). 136(Suppl):S397–S406. doi: 10.1093/infdis/136.supplement_3.s397 606763

[15] Wright PF Thompson J Vaughn WK Folland DS Sell SH. And karzon D.T. trials of influenza A/New Jersey/76 virus vaccine in normal children: an overview of age-related antigenicity and reactogenicity. J Infect Dis (1977). 136(Suppl):S731–S741. doi: 10.1093/infdis/136.supplement_3.s73 606798

[16] Wood JM Schild GC Newman RW Seagroatt V. An improved single-radial-immunodiffusion technique for the assay of influenza haemagglutinin antigen: application for potency determinations of inactivated whole virus and subunit vaccines. J Biol Stand (1977) 5(3):237–47. doi: 10.1016/s0092-1157(77)80008-5 408355

[17] Committee for Proprietary Medicinal Products. Note for guidance on harmonization of requirements for influenza vaccines (1997). Available at: https://www.ema.europa.eu/en/documents/scientific-guideline/note-guidance-harmonisation-requirements-influenza-vaccines_en.pdf (Accessed 15 December 2022).

[18] Levie K Leroux-Roels I Hoppenbrouwers K Kervyn AD Vandermeulen C Forgus S. An adjuvanted, low-dose, pandemic influenza a (H5N1) vaccine candidate is safe, immunogenic, and induces cross-reactive immune responses in healthy adults. J Infect Dis (2008) 198(5):642–9. doi: 10.1086/590913 18576945

[19] Minor PD. Assaying the potency of influenza vaccines. Vaccines (Basel) (2015) 3(1):90–104. doi: 10.3390/vaccines3010090 26344948 PMC4494238

[20] President’s Council of Advisors on Science and Technology (US). Report to the President on reengineering the influenza vaccine production enterprise to meet the challenges of pandemic influenza (2010). Available at: https://www.hsdl.org/?view&did=19766 (Accessed 15 December 2022).

[21] Cuellar-Camacho JL Bhatia S Reiter-Scherer V Lauster D Liese S Rabe JP. Quantification of multivalent interactions between sialic acid and influenza a virus spike proteins by single-molecule force spectroscopy. J Am Chem Soc (2020) 142(28):12181–92. doi: 10.1038/nri.2016.67 32538085

[22] Lerner RA. Combinatorial antibody libraries: new advances, new immunological insights. Nat Rev Immunol (2016) 16(8):498–508. doi: 10.1038/nri.2016.67 27374636

[23] Tung Yep A Takeuchi Y Engelhardt OG. And hufton S.E. broad reactivity single domain antibodies against influenza virus and their applications to vaccine potency testing and immunotherapy. Biomolecules (2011) 11:407. doi: 10.3390/biom11030407 PMC8001348 33802072

[24] Schmeisser F Friedman R Besho J Lugovtsev V Soto J Wang W. Neutralizing and protective epitopes of the 2009 pandemic influenza H1N1 hemagglutinin. Influenza Other Respir Viruses. (2013) 7(3):480–90. doi: 10.1111/irv.12029 PMC5779835 23122228

[25] Schmeisser F Vasudevan A Verma S Wang W Alvarado E Weiss C. Antibodies to antigenic site a of influenza H7 hemagglutinin provide protection against H7N9 challenge. PloS One (2015) 28(10(1)):e0117108. doi: 10.1371/journal.pone.0117108 PMC4309539 25629172

[26] Verma S Soto J Vasudevan A Schmeisser F Alvarado-Facundo E Wang W. Determination of influenza b identity and potency in quadrivalent inactivated influenza vaccines using lineage-specific monoclonal antibodies. PloS One (2017) 12(4):e0175733. doi: 10.1371/journal.pone.0175733 28423025 PMC5396888

[27] Nilsson CE Abbas S Bennemo M Larsson A Hämäläinen MD Frostell-Karlsson A. A novel assay for influenza virus quantification using surface plasmon resonance. Vaccine (2010) 28(3):759–66. doi: 10.1016/j.vaccine.2009.10.070 19857452

[28] Bodle J Verity EE Ong C Vandenberg K Shaw R Barr IG. Development of an enzyme-linked immunoassay for the quantitation of influenza haemagglutinin: an alternative method to single radial immunodiffusion. Influenza Other Respir Viruses (2013) 7(2):191–200. doi: 10.1111/j.1750-2659.2012.00375.x 22583601 PMC5780761

[29] Hashem AM Gravel C Farnsworth A Zou W Lemieux M Xu K. A novel synthetic receptor-based immunoassay for influenza vaccine quantification. PloS One (2013) 8(2):e55428. doi: 10.1371/journal.pone.0055428 23424631 PMC3570553

[30] Khurana S King LR Manischewitz J Coyle EM Golding H. Novel antibody-independent receptor-binding SPR-based assay for rapid measurement of influenza vaccine potency. Vaccine (2014) 32(19):2188–97. doi: 10.1016/j.vaccine.2014.02.049 24613520

[31] Kuck LR Sorensen M Matthews E Srivastava I Cox MM. And rowlen K.L. titer on chip: new analytical tool for influenza vaccine potency determination. PloS One (2014) 9(10):e109616. doi: 10.1371/journal.pone.0109616 25330238 PMC4203742

[32] Schmeisser F Vasudevan A Soto J Kumar A Williams O Weir JP. A monoclonal antibody-based immunoassay for measuring the potency of 2009 pandemic influenza H1N1 vaccines. Influenza Other Respir Viruses (2014) 8(5):587–95. doi: 10.1111/irv.12272 PMC4181825 25087462

[33] Durous L Julien T Padey B Traversier A Rosa-Calatrava M Blum LJ. SPRi-based hemagglutinin quantitative assay for influenza vaccine production monitoring. Vaccine (2019) 37(12):1614–21. doi: 10.1016/j.vaccine.2019.01.083 30773402

[34] Yang D Singh A Wu H Kroe-Barrett R. Comparison of biosensor platforms in the evaluation of high affinity antibody-antigen binding kinetics. Anal Biochem (2016) 508:78–96. doi: 10.1016/j.ab.2016.06.024 27365220

[35] Krause JC Tsibane T Tumpey TM Huffman CJ Basler CF. And crowe J.E. jr. a broadly neutralizing human monoclonal antibody that recognizes a conserved, novel epitope on the globular head of the influenza H1N1 virus hemagglutinin. J Virol (2011) 85(20):10905–8. doi: 10.1128/JVI.00700-11 PMC3187471 21849447

[36] Stöhr K Bucher D Colgate T Wood J. Influenza virus surveillance, vaccine strain selection, and manufacture. Methods Mol Biol (2012) 865:147–62. doi: 10.1007/978-1-61779-621-0_9 22528158

[37] Schmeisser F Jing X Joshi M Vasudevan A Soto J Li X. A novel approach for preparation of the antisera reagent for potency determination of inactivated H7N9 influenza vaccines. Influenza Other Respir Viruses (2016) 10(2):134–40. doi: 10.1111/irv.12365 PMC4746557 26616263

[38] Karlsson R Michaelsson A Mattsson L. Kinetic analysis of monoclonal antibody-antigen interactions with a new biosensor based analytical system. J Immunol Methods (1991) 145(1-2):229–40. doi: 10.1016/0022-1759(91)90331-9 1765656

[39] Bland JM. And altman D.G. measuring agreement in method comparison studies. Stat Methods Med Res (1999) 8(2):135–60. doi: 10.1177/096228029900800204 10501650

[40] Hong M Lee PS Hoffman RM Zhu X Krause JC Laursen NS. Antibody recognition of the pandemic H1N1 influenza virus hemagglutinin receptor binding site. J Virol (2013) 87(22):12471–80. doi: 10.1128/JVI.01388-13 PMC3807900 24027321

[41] World Health Organization. Recommended composition of influenza virus vaccines for use in the 2021 southern hemisphere influenza season (2020). Available at: https://cdn.who.int/media/docs/default-source/emergency-preparedness/global-influenza-programme/recommended-composition-of-influenza-virus-vaccines/202009-recommendation.pdf?sfvrsn=1331fa94_2&download=true (Accessed 21 November 2022).

[42] Kuck LR Saye S Loob S Roth-Eichhorn S Byrne-Nash R. And rowlen K.L. VaxArray assessment of influenza split vaccine potency and stability. Vaccine (2017) 35(15):1918–25. doi: 10.1016/j.vaccine.2017.02.028 28262335

[43] Arunachalam AB Post P Rudin D. Unique features of a recombinant haemagglutinin influenza vaccine that influence vaccine performance. NPJ Vaccines (2021) 6(1):144. doi: 10.1038/s41541-021-00403-7 34857771 PMC8640007

[44] Ekimov A Arunachalam AB Blake T Bodle J Couzens L Dubey S. Assessing the stability-indicating properties of alternative potency assays for inactivated influenza vaccine. Vaccine (2023). doi: 10.1016/j.vaccine.2023.06.051 37344260

